# An alternative splice isoform of mouse CDK5RAP2 induced cytoplasmic microtubule nucleation

**DOI:** 10.1016/j.ibneur.2022.09.004

**Published:** 2022-09-15

**Authors:** Akari Nakamura, Mami Ikeda, Seina Kusayanagi, Kensuke Hayashi

**Affiliations:** Department of Materials and Life Sciences, Faculty of Science and Technology, Sophia University, Tokyo, Japan

**Keywords:** CKD5RAP2, cyclin-dependent kinase 5 regulatory subunit-associated protein 2, CM1, centrosomin motif 1, DMEM, Dulbecco’s Modified Eagle’s Medium, FBS, fetal bovine serum, γTuRC, γ-tubulin ring complex, HBSS, Hanks' Balanced Salt Solution, IB, immunoblotting, IP, immunoprecipitation, MT, microtubule, MZT2, MOZART2, NSD, nonsense-mediated mRNA decay, PBS, phosphate-buffered saline, PCR, polymerase chain reaction, Centrosome, MZT2, γTuRC, Dendrite growth, Neuron differentiation, Microcephaly

## Abstract

The centrosome lacks microtubule (MT)-nucleation activity in differentiated neurons. We have previously demonstrated that MTs were nucleated at the cytoplasm of mouse neurons. They are supposed to serve seeds for MTs required for dendrite growth. However, the factors that activate the cytoplasmic γ-tubulin ring complex (γTuRC) are unknown. Here we report an alternative splicing isoform of cyclin-dependent kinase 5 regulatory subunit-associated protein 2 (CKD5RAP2) as a candidate for the cytoplasmic γTuRC activator. This isoform lacked exon 17 and was expressed predominantly in the brain and testis. The expression was transient during the development of cortical neurons, which period coincided with the period we reported cytoplasmic MT nucleation. This isoform resulted in a frameshift and generated truncated protein without a centrosomal localization signal. When this isoform was expressed in cells, it localized diffusely in the cytoplasm. It was co-immunoprecipitated with γ-tubulin and MOZART2, suggesting that it can activate cytosolic γTuRCs. After cold-nocodazole depolymerization of MTs and subsequent washout, we observed numerous short MTs in the cytoplasm of cells transfected with the cDNA of this isoform. The isoform-overexpressing cells exhibited an increased amount of MTs and a decreased ratio of acetylated tubulin, suggesting that MT generation and turnover were enhanced by the isoform. Our data suggest the possibility that alternative splicing of CDK5RAP2 induces cytoplasmic nucleation of MTs in developing neurons.

## Highlights

• Developing cortical neurons transiently express an alternative splicing isoform of CDK5RAP2.
• This isoform was localized at the cytoplasm and associated with γTuRCs.
• This isoform induced nucleation of microtubules at the cytoplasm.
• We propose that this isoform generates microtubule seeds to grow dendrites.

## Graphical Abstract

**Figure:**
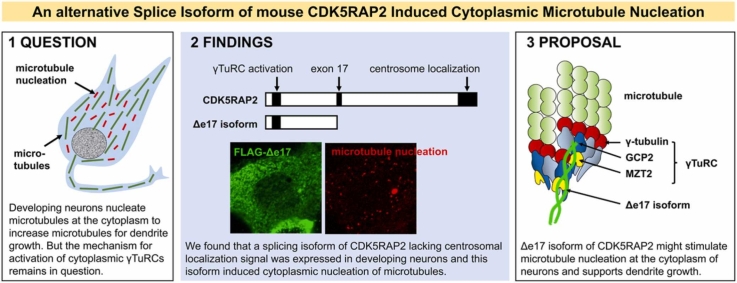

## 1 Introduction

The function of the brain depends to some extent on the number of synapses, which depends on the length and complexity of the dendrites. Therefore, the establishment of the intricate configuration of dendrites is the basic foundation for the development of the brain. Microtubule cytoskeleton (MT) is essential to support dendrite morphology and must increase in length and number during the dendrite growth (Delandre et al., 2016, Lasser et al., 2018, Luders, 2021). The de novo formations of microtubules are performed by nucleation of MTs from tubulin dimers initiated by the γ-tubulin ring complex (γTuRC) (Kollman et al., 2011). In general, γTuRC distributes throughout the cytoplasm, but only at the centrosome, it is activated by interaction with the γTuRC-activating proteins that are associated with the centrosome. However, the centrosomes of neurons have been reported to lose γTuRC-activating proteins and lose the ability to nucleate MTs (Leask et al., 1997, Stiess et al., 2010, Yonezawa et al., 2015). Various studies have been done to elucidate the nature of non-centrosomal MT nucleation in mammalian and in non-mammalian neurons (Wilkes and Moore, 2020, Luders, 2021, Weiner et al., 2021), including an extensive live-image analysis on MT nucleation at growth cones of C. elegance (Liang et al., 2020). Some key molecules for non-centrosomal MT nucleation in mammalian neurons have been proposed, such as the HAUS complex (Sanchez-Huertas et al., 2016, Cunha-Ferreira et al., 2018) and TPX2 activated by Ran (Chen et al., 2017b).

We reported that MT nucleation occurs in the cytoplasm of developing neurons (Yamada and Hayashi, 2019). After depolymerizing preexisting MTs and subsequent washout and incubation, many short MTs were generated in the cytoplasm of primary cultured mouse neurons. Gamma-tubulin and MOZART2 (MZT2) were immunologically detected at one end of these MTs, suggesting that the cytoplasmic γTuRCs were activated. The MT-nucleation in the cytoplasm of cell bodies and dendrites explains well the random polarity of MTs in dendrites, and can also explain how MTs rapidly increase in number when dendrites start to grow (Yu et al., 2000, Yamada and Hayashi, 2019). However, the mechanisms for activation of cytoplasmic γTuRC in neurons we observed in our previous report are unknown.

Recently, the molecular structure of γTuRC has been unveiled by cryo-electron microscopy. It was revealed that the isolated γTuRC has low nucleation efficiency and this is because of the mismatch between the arrangement of γ-tubulins around the ring and the symmetric arrangement of αβ-tubulin dimers of the MT (Consolati et al., 2020, Liu et al., 2020, Wieczorek et al., 2020b). To acquire higher nucleation efficiency, γTuRC should be stimulated by other proteins, such as TPX2, TOG domain proteins, and centrosomin motif 1 (CM1) domain proteins (Tovey and Conduit, 2018). Among them, TPX2 and TOG domain proteins stimulate nucleation by stabilizing early MT nucleation intermediates without detectable direct interaction with γTuRC (Roostalu et al., 2015). In contrast, one of the CM1 domain proteins, cyclin-dependent kinase 5 regulatory subunit-associated protein 2 (CDK5RAP2) was shown to interact directly with γTuRC (Choi et al., 2010). The N-terminal CM1 domain of CDK5RAP2 makes a tripartite complex with MZT2 and GCP2 of γTuRC, as revealed by cryo-electron microscopy (Wieczorek et al., 2020a). CDK5RAP2 has been implicated in γTuRC activation in most of the MT-organizing centers including the centrosome and the Golgi apparatus (Kollman et al., 2011, Ide et al., 2021). CDK5RAP2 homolog, Centrosomin, localizes to the Golgi outposts in Drosophila sensory neurons and is involved in MT nucleation in the dendrites (Yalgin et al., 2015; Feng et al., 2021). In humans, CDK5RAP2 is one of the causal genes of autosomal recessive primary microcephaly, indicating that it is important for neuronal development (Bond et al., 2005, Kraemer et al., 2011, Zaqout and Kaindl, 2022). Thus, it would be reasonable that CDK5RAP2 stimulates MT-nucleation at the cytoplasm in developing neurons. This is, however, impossible, as long as CDK5RAP2 is strongly stuck to the centrosome through its C-terminal centrosome targeting domain.

During the differentiation of neural stem cells to neurons, various centrosome proteins are lost from the centrosome (Ohama and Hayashi, 2009, Stiess et al., 2010, Yonezawa et al., 2015). For example, ninein, a protein that anchors MTs to the centrosome, changes its localization from the centrosome to the cytoplasm during neuron differentiation (Ohama and Hayashi, 2009). This change in localization is due to selective splicing of ninein mRNA, skipping the exon containing the centrosome-binding domain (Zhang et al., 2016). In addition to ninein, many other cytoskeletal proteins were shown to undergo splicing changes during neuronal differentiation and to play important roles in neuronal differentiation (Zhang et al., 2016).

Therefore, we reasoned that CDK5RAP2 also undergoes splicing changes during neuronal differentiation and that an isoform without a centrosomal localizing signal is expressed in developing neurons. We searched the nucleotide database for mouse CDK5RAP2 splicing isoforms and examined their expression. We found a neuron-specific splicing isoform of which expression was transient during neuronal differentiation. This isoform lacked a centrosome binding domain. It localized to the cytoplasm and induced MT nucleation in the cytoplasm, demonstrating the ability of this isoform to activate cytoplasmic γTuRCs. These data suggest that the cytoplasmic MT nucleation in developing neurons is caused by alternative splicing of CDK5RAP2.

## Experimental procedures

### Animals

ICR mice were obtained from Japan SLC, Inc. (Shizuoka, Japan). They were euthanized with CO_2_. All procedures were conducted according to the Guide for the Care and Use of Laboratory Animals (NIH publications No. 80–23) and the guidelines of the Animal Experiment Committee of Sophia University.

### Primary antibodies

Antibodies and their dilutions were as follows: rabbit polyclonal antibody against pericentrin (Covance #923701, Burlington, NC) [1:500], rabbit polyclonal antibody against CDK5RAP2 (MILLIPORE #06–1398, Burlington, MA) [1:500], mouse monoclonal antibody against α-tubulin (Sigma-Aldrich #T5168, St. Louis, MO) [1:500], rabbit polyclonal antibody against α-tubulin (Thermo Fisher Scientific #RB-9281, Waltham, MA) [1:200], mouse monoclonal antibody against acetylated tubulin (Sigma-Aldrich #T6793) [1:500], goat polyclonal antibody against γ-tubulin (Santa Cruz #sc-7396, Santa Cruz, CA) [1:500], mouse monoclonal antibody against FLAG (clone M2; Sigma-Aldrich) [1:1000] and mouse monoclonal antibody against GM130 (BD Biosciences #610822, Franklin Lakes, NJ) [1:400]. The generation of rabbit antiserum against MZT2 [1:200] is described in (Yamada and Hayashi, 2019). All antibodies were diluted with 10% fetal bovine serum (FBS) and 0.1% Triton X-100 in phosphate-buffered saline (PBS).

### cDNA constructs

Δe17 cDNA expressing vector was constructed by PrimeSTAR Mutagenesis Basal Kit (Takara Bio, Shiga, Japan) from mouse FLAG-tagged CDK5RAP2 cDNA expression plasmid (addgene #106911, Watertown, MA) and an exon 16–18 spanning primer set (Table 1). GFP-tagged CDK5RAP2 expression vector was constructed by insertion of PCR-amplified ORF of the above construct into *Eco*RI-*Kpn*I site of pEGFP-C1 vector (Clontech/ Takara Bio).

**Table 1:** PCR primers used in this study.

e16-18 spanning forward	AGTCGAGAAGGTCAGTGACCTCATACAGCTT
e16-18 spanning reverse	AGGTCACTGACCTTCTCGACTCCGAATCTCAC
Pr1	CCTACCGGAACCTGCAGAAG
Pr2	CCAATTGGAGCATTTTTCTCA
Pr3	CGGAGTCGAGAAGGTCAGTG
Pr4	CTGTCACTTCCCTGGAAACC
Pr5	TCTTCAGAGGCACCAGGAGT
GAPDH forward	TGTAGGCCATGAGGTCCACCA
GAPDH reverse	TGTAGGCCATGAGGTCCACCA

### Primary culture of cortical neurons

The primary culture of neurons was prepared as described elsewhere (Yamada and Hayashi, 2019). Briefly, cerebral cortices were obtained from mice on embryonic day 16.5 and were dissociated by treatment with 9 U/mL papain (Worthington Biochemical, Lakewood, NJ) for 15 min at 37 °C, followed by trituration with a pipette. Cells were plated on plastic dishes that were treated with 0.0025% poly-L-lysine (Sigma-Aldrich), followed by treatment with 10% horse serum in Hanks' Balanced Salt Solution (HBSS). They were cultured in NeuroBasal medium supplemented with B27 (Invitrogen, Carlsbad, CA). Neuron-free astrocyte culture was prepared from dissociated cells of embryonic cortices. Cells were grown in Dulbecco’s Modified Eagle’s Medium (DMEM) supplemented with 10% FBS and penicillin /streptomycin. During more than 10-days of culture, neurons were removed by repetitive shaking of the culture bottles.

### RT-PCR

**Fig. 1:**
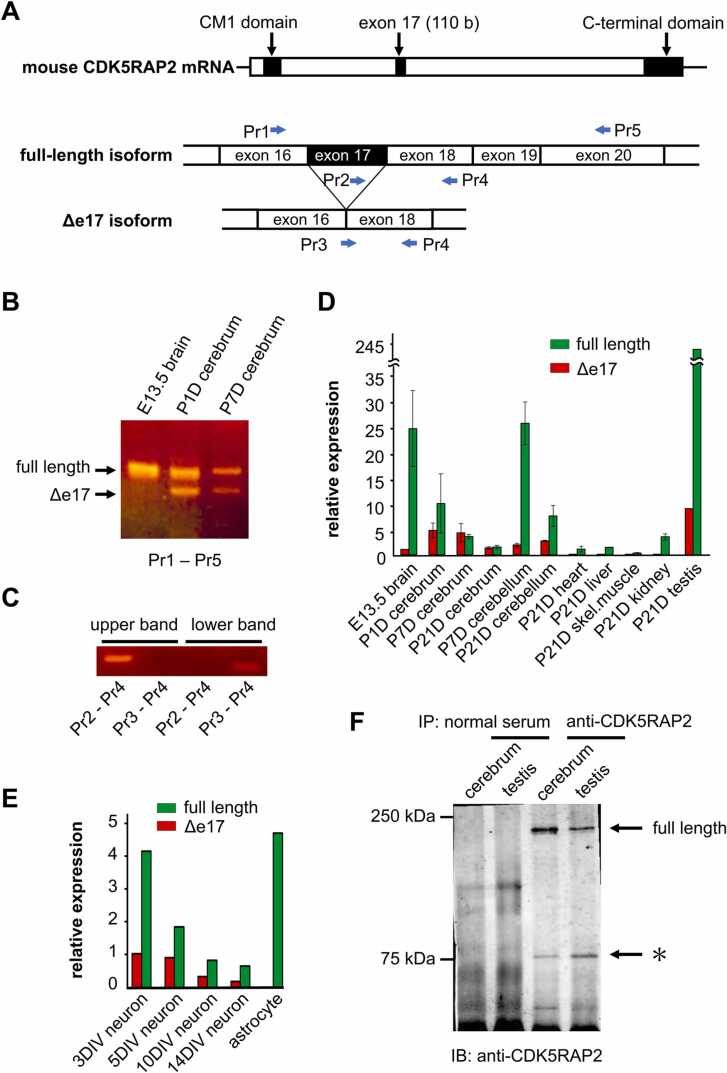
An alternative splice isoform of CDK5RAP2. (A) mRNA structure of mouse CDK5RAP2. CM1 domain refers to the centrosomin N-terminal motif 1, which binds to and activates γTuRC. The C-terminal domain is responsible for the localization of CDK5RAP2 at the centrosome and at the Golgi apparatus. Primers used for RT-PCR were shown. (B) RT-PCR analysis on the exon 17 skipping. Total RNA from embryonic day 13.5 brain, postnatal day 1 (P1D), and postnatal day 7 cerebrum were analyzed with primers flanking exon 17 (Pr1 and Pr5). Skipping of exon 17 (Δe17) was detected after P1D. (C) Amplicons with primer sets used for real time-RT-PCR shown in (D). DNAs were eluted from the upper and the lower bands of the gel shown in (B) and were amplified with primers specific to each isoform. (D) Quantification of the expression of CDK5RAP2 isoforms measured with real-time RT-PCR. The full-length isoform was amplified with primer set Pr2 and Pr4, and the Δe17 isoform was amplified with primer set Pr3 and Pr4. Error bars are S. E. (E) Real-time PCR analysis on the change in the isoform expression during the culture of primary cortical neurons. DIV stands for days in vitro. (F) Detection of CDK5RAP2 proteins in tissues. Lysates from the P7D cerebrum and P21D testis were immunoprecipitated with anti-CDK5RAP2 antibody and immunoblotted with the same antibody. The star indicates the band with a molecular weight close to the calculated molecular weight of Δe17 isoform.

Total RNA was extracted with the Fast Pure RNA Kit (Takara Bio). RNAs were quantified with a BioPhotometer (Eppendorf, Hamburg, Germany), and their integrity was confirmed by gel electrophoresis. Two µg of total RNAs were used for cDNA synthesis with random hexamer and PrimeScript RT Master Mix (Takara Bio) according to the manufacturer's protocol. For semi-quantitative PCR, Prime STAR HS (Takara Bio) and primer sets Pr1 and Pr5 (see Fig. 1A and Table 1) were used. For real-time PCR, TB Green Premix Ex *Taq*II (Takara Bio) and primer sets Pr3, Pr4 and Pr5 (see Fig. 1A and Table 1) were used. The fluorescence was quantified with QuantStudio 3 instrument (ABI 7500; Applied Biosystems, Foster City, CA). Relative mRNA levels for each isoform were calculated by the 2^ΔΔCt method using GAPDH (primer set is listed in Table 1) as an internal control.

### Cell culture and gene transfer

HEK293T, COS and Neuro2A cells were cultured in DMEM supplemented with 10% FBS and penicillin /streptomycin. Transfection of cells with plasmids was performed with Polyethyleneimine ''Max'' (Mw 40000) (Polysciences, Inc; Warrington, PA).

### Immunoprecipitation and Western blotting

For CDK5RAP2 isoform detection, tissues or cells were lysed with RIPA buffer (50 mM Tris-HCl pH7.4, 150 mM NaCl, 1% Triton X-100, 1 mM EDTA) supplemented with 1 mM phenylmethylsulfonyl fluoride, 1 μg/mL aprotinin and 1 μg/mL leupeptin. The homogenates were centrifuged at 15,000 rpm for 30 min. Ten μL of a slurry of Protein G Sepharose 4 Fast Flow (GE Healthcare Bio-Sciences, Uppsala, Sweden) were premixed with 1 μg of anti-CDK5RAP2 antibody for 2 h, washed 3 times with RIPA buffer, and incubated with 200 μL of the tissue or cell lysate at 4 °C for 4 h. For assays of γTuRC binding, lysates from HEK293T or COS cells transfected with FLAG-tagged CDK5RAP2 isoforms were incubated with Protein G Sepharose that was premixed with an anti-FLAG antibody. The Sepharose beads were washed 3 times with RIPA buffer and were boiled in SDS sample buffer.

Proteins were applied on SDS-PAGE and were transferred to Immobilon FL membranes (Millipore). Membranes were immunostained and analyzed with the Odyssey system (LI-COR Biosciences, Lincoln, NE). Alexa Fluor 680 labeled anti-rabbit and anti-goat IgG (Thermo Fisher Scientific) [1:5000] was used for the secondary antibody.

### MT regrowth experiment

To depolymerize MTs, 10 µg/mL nocodazole (FUJIFILM Wako, Osaka, Japan) was added to the culture medium, and the cells were incubated for 60 min at 37 °C. They were then transferred on ice and kept for 120 min. After washing with ice-cold HBSS 5 times, the medium was replaced with a 37 °C medium without nocodazole and incubated for 30 s at 37 °C. Then, cells were quickly fixed and immunostained as described below. To analyze the number of cytoplasmic regrown MTs, we collected images of an optical slice that included the centrosome of randomly selected FLAG-positive cells with a laser confocal microscope (LSM700, Carl Zeiss, Oberkochen, Germany). MTs that were not associated with the centrosome were counted for each cell. The data were collected from three independent experiments.

### Immunostainings and observation

**Fig. 2:**
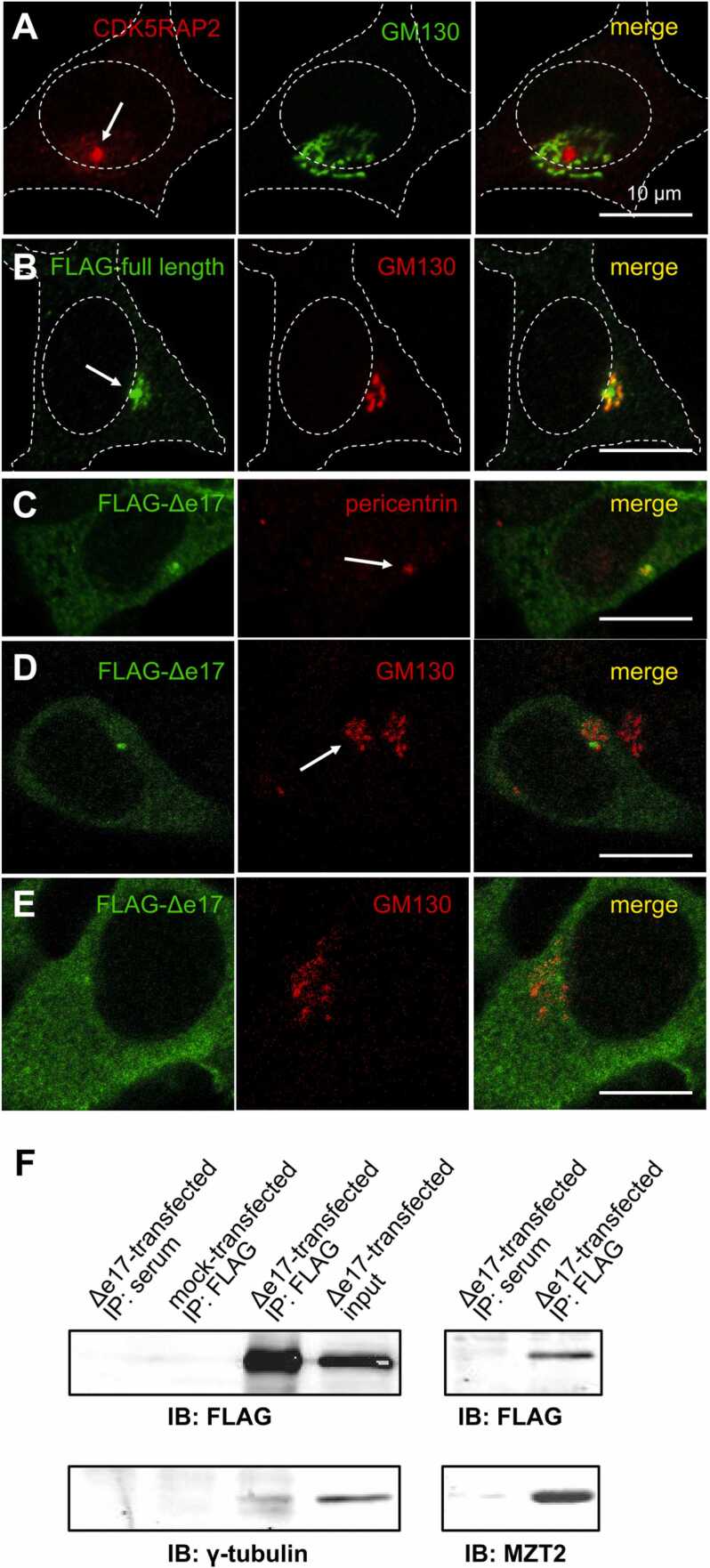
Δe17 isoform localized diffusely in cytoplasm and binds to γTuRC in HEK293T cells. Cells were observed with a laser confocal microscope. (A) Immunostaining with anti-CDK5RAP2 antibody and GM130 antibody (Golgi marker). Endogenous CDK5RAP2 localized intensely at the centrosome (arrow) and weakly on the Golgi apparatus. (B) Anti-FLAG immunostaining of FLAG-tagged full-length CDK5RAP2. The full-length isoform localized intensely at the centrosome (arrow) and weakly on the Golgi apparatus. (C) Localization of FLAG-tagged Δe17 isoform in cells expressing low level of the isoform. FLAG immunostaining showed diffuse localization of the isoform in the cytoplasm as well as weak localization at the centrosome (arrow). (D) Localization of FLAG-tagged Δe17 isoform in cells expressing low level of the isoform. FLAG immunostaining did not show obvious localization of the isoform at the Golgi apparatus (arrow). (E) Localization of FLAG-tagged Δe17 isoform in cells expressing a high level of the isoform. FLAG immunostaining showed diffuse and even localization in the cytoplasm. (F) Interaction of Δe17 isoform with γTuRC. Lysates of cells transfected with FLAG-tagged CDK5RAP2 isoforms were immunoprecipitated with FLAG antibody and were immunoblotted with anti-γ-tubulin antibody and with anti-MZT2 antibody.

**Fig. 3:**
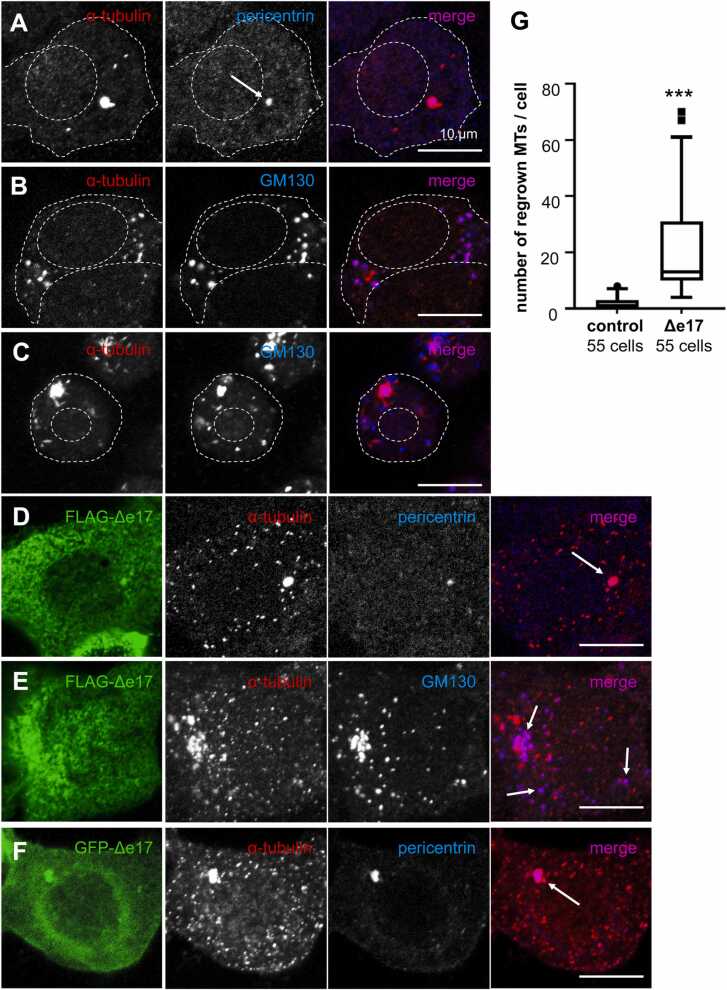
MT regrowth experiments. (A, B) HEK293T cells were treated with cold-nocodazole, washed out, and incubated to allow MT regrowth. Regrown MTs were detected by α-tubulin antibody. Pericentrin antibody stained the centrosome and GM130 antibody stained the Golgi apparatus. Cells were observed with a laser confocal microscope. MT regrowth was observed at the centrosome (arrow in A) and the Golgi apparatus (B). (C) Neuro2A cells were treated as in (B) to show similar regrowth pattern of MTs as HEK293T cells. (D, E) Triple immunostaining of cells for FLAG, regrown MTs (α-tubulin), and the centrosome (pericentrin) or the Golgi apparatus (GM130). Cells expressing FLAG-tagged Δe17 isoform exhibited evenly distributing regrown MTs throughout the cytoplasm. Some of them were colocalized with the centrosome (arrow in D) or the Golgi apparatus (arrows in E), but others were not associated with the centrosome nor with the Golgi apparatus. (F) Neuro2A cells transfected with GFP-tagged Δe17 isoform. A similar distribution of regrown MTs to (D) was observed. Arrow indicates the centrosome. (G) The number of regrown MTs was counted in control cells and FLAG-tagged Δe17 isoform-transfected HEK293T cells. The graph is shown with the tukey method of box and whisker plot. ***p < 0.001.

**Fig. 4:**
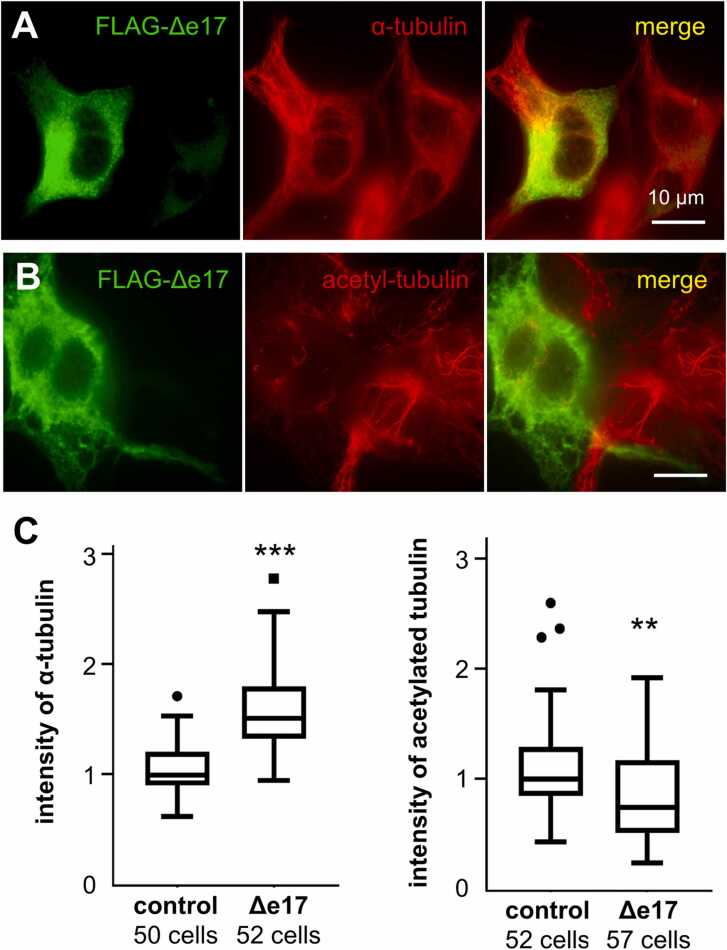
Change in the MT amount and MT dynamics in Δe17 isoform-expressing HEK293T cells. (A) Δe17 isoform-expressing cells were fixed in the presence of Triton X-100 and Taxol to washout free tubulin dimers before being immunostained with anti-α-tubulin antibody. Fluorescence was photographed under a conventional fluorescent microscope. (B) Δe17 isoform-expressing cells were immunostained with an anti-acetylated-tubulin antibody. (C) Fluorescence was photographed under a conventional fluorescent microscope and the fluorescence intensity was measured and statistically analyzed. Graphs were shown with the tukey method of box and whisker plot. ***p < 0.001, **p < 0.01.

For examination of isoform localization in cells (Fig. 2), cells were fixed with 4% paraformaldehyde in 0.1 M sodium phosphate buffer (pH 7.4) for 2 min and post-fixed in methanol at − 20 °C for 8 min. For MT regrowth experiments (Fig. 3) and staining of acetylated or non-acetylated MTs (Fig. 4), cells were fixed with 4% paraformaldehyde, 1% Triton X-100, and 10 µM Taxol in PHEM (60 mM PIPES, 10 mM EGTA, 25 mM HEPES, 2 mM MgCl_2_, pH 6.9) at 37 ℃ for 2 min. Then, they were post-fixed with methanol at − 20 °C for 8 min. Fixed cells were treated with 0.1% Triton X-100 in PBS for 30 min and subsequently with 10% FBS and 0.1% Triton X-100 in PBS for 1 h. They were then incubated with the primary antibody for 1 h, washed in 0.1% Triton X-100 in PBS three times, and then incubated with Alexa Flour 488, 568, or 647 donkey anti-mouse, rabbit, or goat IgG (Thermo Fisher Scientific) [1:200] for 1 h. These antibodies were diluted in 10% FBS and 0.1% Triton X-100 in PBS. After washing three times, cells were observed using either a laser confocal microscope (LSM700, Carl Zeiss) or a conventional fluorescent microscope (AxioVert200M, Carl Zeiss). Alexa Flour 647 staining was shown in blue.

To quantify the amount of MTs and acetylated MTs, transfected cells were stained with an antibody against α-tubulin or with an antibody against acetylated tubulin. Images of immuno-fluorescence of randomly selected FLAG-positive cells were collected with a conventional fluorescent microscope (AxioVert200M, Carl Zeiss) with a fixed exposure time. ImageJ software was used for measurement of the mean value of fluorescence for each cell. The data were collected from three independent experiments.

### Statistical analysis

Statistical analysis was performed in GraphPad Prism (GraphPad Software). Significance was tested by Student's t-test and indicated as follows: * **p < 0.001, * *p < 0.01.

## Results

### Alternative splicing isoform of mouse CDK5RAP2

The N-terminal CM1 domain of CDK5RAP2 is a γTuRC-binding domain and the C terminal domain is responsible for the localization of CDK5RAP2 at the centrosome or the Golgi apparatus (Fig. 1A) (Wang et al., 2010). BLAST search of the mouse CDK5RAP2 sequence revealed three types of splicing isoforms. One skipped exons 4 and 5, another skipped exon 12, and the other skipped exon 17. We examined the expression of these isoforms by RT-PCR using total RNA from primary cultured cortical neurons and neuron-free astrocytes and found that only the isoform that skips exon 17 (Δe17 isoform) was specific to neurons. RT-PCR analysis of brains during development revealed that the expression of full-length isoform decreased during the development, but that Δe17 isoform began to be expressed immediately after birth (Fig. 1B).

To quantify the expression of splicing isoforms, primers specific for full-length isoform and Δe17 isoform (Pr2-Pr4 pair and Pr3-Pr4 pair, respectively) were designed. The isoform specificity of these primer pairs was confirmed by PCR using extracted cDNA from the upper and lower bands of Fig. 1B as the templates (Fig. 1C). During cerebral development, the expression of the full-length isoform decreased from embryonic day 13.5 (E13.5) to postnatal day 21 (P21D) as reported by (Issa et al., 2013), while the expression of Δe17 isoform increased rapidly on P1D (Fig. 1D). The expression of Δe17 isoform was very low in the heart, liver, skeletal muscle, and kidney. In primary cultured cortical neurons, the expression of Δe17 isoform was high on the 3rd day and the 5th day in vitro (DIV) and became lower on 10DIV (Fig. 1E). This time course coincides with the time course in which MT nucleation in the cytoplasm was observed in our previous study (Yamada and Hayashi, 2019).

Exon 17 skipping gives rise to a frameshift and results in a stop codon in exon 18. In general, some but not all mRNAs bearing premature termination codons are degraded by nonsense-mediated mRNA decay (NSD) (Supek et al., 2021). In the present case, a considerable amount of Δe17 isoform was detected, indicating that the mRNA escaped from NSD for unknown reasons. To determine whether this transcript was translated, we performed Western blotting. Since only vague bands were detected in Western blotting of tissue homogenates, we immunoprecipitated proteins from tissue lysates with antibodies to CDK5RAP2, which epitope was the N-terminus of CDK5RAP2. The precipitates were subjected to SDS-PAGE, and bands were detected with the same antibody. In addition to the full size CDK5RAP2 band, we found bands with a smaller molecular weight in the P7D cerebrum and P21D testis (Fig. 1F, star). The band size was close to the calculated molecular weight of Δe17 isoform (70.2 kDa), and the band was not detected in the control lane, suggesting that the band represents the Δe17 isoform.

### Δe17 isoform localized in the cytoplasm

Because the truncated product of the Δe17 isoform lacked the centrosome-binding domain at the C-terminus, it was expected that this protein would localize at the cytoplasm. We examined the subcellular localization of Δe17 isoform by transfecting FLAG-tagged Δe17 isoform-expressing plasmid into HEK293T cells. In un-transfected HEK293T cells, endogenous CDK5RAP2 localized predominantly at the centrosome, with a small amount at the Golgi apparatus (Fig. 2A). FLAG-tagged full-length isoform localized mainly at the centrosome and weakly at the Golgi apparatus (Fig. 2B). In contrast, FLAG-tagged Δe17 isoform localized weakly at the centrosome and diffusely at the cytoplasm even in weakly expressing cells (Fig. 2C, D). Also in cells with an intense expression, the Δe17 isoform was distributed uniformly in the cytoplasm (Fig. 2E). To examine whether the cytoplasmic Δe17 isoform protein binds to γTuRC, we immunoprecipitated the lysate of Δe17 isoform-expressing cells with FLAG antibody and found that γ-tubulin and MZT2 were co-immunoprecipitated (Fig. 2F). These results suggest that the cytoplasmic Δe17 isoform binds to cytoplasmic γTuRC.

### Δe17 isoform nucleates MTs in the cytoplasm

We investigated whether the Δe17 isoform stimulates cytoplasmic nucleation of MT. We performed MT-regrowth experiments, in which preexisting MTs were depolymerized with cold-nocodazole, and de novo formation of MTs after nocodazole-washout and incubation were detected with α-tubulin antibody. In un-transfected HEK293T cells, regrown MTs were predominantly observed at the centrosome, with a small number at the cytoplasm (Fig. 3A). These cytoplasmic MTs co-localized with the Golgi apparatus, which were fragmented by cold-nocodazole treatment (Fig. 3B). A similar regrowth pattern of MTs was observed in another cell type, Neuro2A cells (Fig. 3C). In cells expressing Δe17 isoform, a considerably large number of short MTs were detected at the cytoplasm in addition to the centrosome (Fig. 3D). They were distributed evenly throughout the cell. Some of these cytoplasmic short MTs co-localized with the Golgi fragments (arrows in Fig. 3E), but most of them were not associated with the Golgi fragments (Fig. 3E). A similar cytoplasmic MT nucleation was observed in Neuro2A cells transfected with GFP-tagged Δe17 isoform (Fig. 3F), indicating that induction of MT nucleation by Δe17 isoform was not specific to HEK293T cells and was not altered with protein tag. The number of cytoplasmic MTs per cell was significantly higher in Δe17 isoform-expressing cells than in mock-transfected control cells (Fig. 3G). These results indicate that the Δe17 isoform activates cytoplasmic γTuRC.

### Overexpression of Δe17 isoform increased MT amount and elevated MT turnover

Stimulation of cytoplasmic MT nucleation by Δe17 isoform led us to examine whether overexpression of Δe17 isoform increases the total amount of MTs in cells. Thus, cells expressing Δe17 isoform were fixed in the presence of Triton-Taxol to remove free tubulin dimers in the cytoplasm, and then MTs were stained with antibodies to α-tubulin (Fig. 4A). Measurement of the fluorescence intensity revealed that the expression of Δe17 isoform significantly increased the MT amount in the cell (Fig. 4C).

MTs are decorated with a variety of posttranslational modifications overtime after polymerization (Yu et al., 2015). Acetylation occurs primarily on microtubules but not on tubulin, and it is abundant on stable microtubules found in cilia and centrioles, as well as on long-lived cytoplasmic microtubules with slow dynamics (Bulinski et al., 1988, Webster and Borisy, 1989). Tubulin acetyltransferase acetylates MT stochastically and functions as a slow clock for microtubule lifetimes (Szyk et al., 2014). When MT nucleation is enhanced by Δe17 isoform expression, it is expected that the amount of newly formed MTs will increase, and thus the ratio of long-lived MTs will decrease. To address this possibility, HEK293T cells expressing Δe17 isoform were treated with Triton-Taxol as described above and then stained with antibodies for acetylated tubulin (Fig. 4B). Measurement of the fluorescence revealed that acetylated MTs significantly decreased (Fig. 4 C), suggesting that the overexpression of Δe17 isoform increased dynamic MTs.

## Discussion

In our previous study, we reported that MTs were nucleated at the cytoplasm in developing cortical neurons (Yamada and Hayashi, 2019). Gamma-tubulin and MZT2 were immunologically detected at one end of the nucleated short MTs, indicating that cytoplasmic γTuRCs were activated (Yamada and Hayashi, 2019). But the mechanisms for the activation of cytoplasmic γTuRC are unknown. In the present study, we report an alternative splicing isoform of CDK5RAP2, which expression was tissue-specific. Introducing this isoform in cells stimulated cytoplasmic MT nucleation. We propose the idea that this splicing isoform of CDK5RAP2 activates cytoplasmic γTuRCs in cortical neurons.

We BLAST searched for splicing isoforms of mouse CDK5RAP2 and investigated their expression pattern. The expression pattern of the Δe17 isoform attracted our interest in the following two respects. First, the expression of this isoform was tissue-specific. It was high in the brain and testis, but low in the heart, liver, and skeletal muscle. Although several splicing isoforms besides exon 17 exclusion have been reported in mice and humans, none of them are expressed predominantly in the brain. For example, the alternatively spliced human CDK5RAP2 lacking exon 32 is not expressed in the brain (Kim et al., 2011). The equivalent splicing variation is not even found in mice (Park et al., 2015). Instead, there have been reported two other splicing isoforms in mouse CDK5RAP2. One is carrying additional axon 3a between exon 3 and exon 4 (Kraemer et al., 2015). This inclusion causes a frameshift at the beginning of the CM1 domain. The second isoform is a transcript from an alternative start site before exon 7 (Kraemer et al., 2015). This variant encodes shorter CDK5RAP2 protein lacking the N-terminus CM1 domain. Although these two isoforms do not have a functional CM1 domain, they are at least in part accountable for the lack of phenotype in the CDK5RAP2 exon 3-deleted mutant mice (Kraemer et al., 2015). The expression pattern and the functions of these truncated CDK5RAP2 proteins are not known. Predominant expression of Δe17 isoform in neurons and the presence of functional CM1 domain in this isoform suggest a certain role of this isoform in the formation of the MT network in neurons.

The second respect in which the expression of this isoform is striking is that it starts after the differentiation of cortical neurons. Transcriptome comparison between undifferentiated neural progenitors and differentiated neurons has revealed hundreds of alternative exon usages during the differentiation of cortical neurons (Zhang et al., 2016). Alternative splicing preferentially regulates genes encoding cytoskeletal proteins. Splicing factors were identified to antagonistically govern the neural progenitor-to-neuron transition by regulating neuron-specific exons (Zhang et al., 2016). This means that skipping of exon 17 of CDK5RAP2 is one of the large-scale alternative splicing events during neuronal differentiation. Moreover, it is noticeable that the transient expression of the Δe17 isoform coincides with the transient occurrence of MT nucleation in the cytoplasm of cultured cortical neurons (Yamada and Hayashi, 2019).

We found that the Δe17 isoform distributed evenly throughout the cytoplasm. This is quite different from the previously reported localization of full-length CDK5RAP2, which generates large aggregates in the cytoplasm when expressed at a high level in cells (Fong et al., 2008). In such cells, γTuRCs are recruited onto the aggregates and MT nucleation occurs from the aggregates. In contrast, MT nucleation in Δe17 isoform-expressing cells was evenly distributed throughout the cytoplasm without any tendency to gather. This pattern is similar to the distribution of MT nucleation induced by expressing a short fragment of the CM1 region of CDK5RAP2 (Choi et al., 2010). We suppose the free-floating Δe17 isoform bind to free-floating γTuRC in the cytoplasm, although we have not yet excluded the possibility if the Δe17 isoform is bound to membrane structures such as mitochondria, which serve MT nucleation sites in Drosophila testis (Chen et al., 2017a). In immunohistochemical studies of CDK5RAP2 during the development of mouse cerebral cortex, CDK5RAP2 has been localized at the centrosome in neuronal progenitors but becomes undetectable in neurons upon differentiation despite the prolonged mRNA expression (Issa et al., 2013, Yonezawa et al., 2015). The reason why the Δe17 isoform has not been immunohistochemically detected at the cytoplasm of neurons maybe because its limited expression and diffuse localization make it difficult to be detected.

CDK5RAP2 is also known as a causative gene for autosomal recessive primary microcephaly. Dysfunction of CDK5RAP2 is thought to cause premature differentiation of neural stem cells and depletion of progenitors (Buchman et al., 2010). These are the results of abnormal cell divisions of neural stem cells (Bond et al., 2005). CDK5RAP2 plays a role in centriole engagement and cohesion (Graser et al., 2007, Barrera et al., 2010), chromosome segregation (Lizarraga et al., 2010), and spindle orientation (Hanafusa et al., 2015), resulting in the shift of the axis of cell divisions of neural stem cells (Lancaster et al., 2013). Apart from the functions in the generation of neural progenitors, the functions of CDK5RAP2 in neurite growth is not well studied. Interestingly, one CDK5RAP2 mutant mouse, designated as CDK5RAP2^an^ according to the name of mutant mouse *Hertwig’s* anemia, showed reduced dendritic complexity in the adult neocortex, in addition to the reduced thickness of the cortex (Zaqout et al., 2019). Since exon 4 is skipped in the CDK5RAP2 ^gen^e, and the CM1 domain is disrupted in the protein (Lizarraga et al., 2010), γTuRC activation by CDK5RAP2 is likely required for dendrite growth. The involvement of CDK5RAP2 in cell process formation is also reported in astrocytes. CDK5RAP2 was somehow localized at the cytoplasm of the glial process, and MT nucleation by cytoplasmic CDK5RAP2 is thought to support the growth of glial processes (Kang et al., 2020).

In conclusion, we provide evidence that the CDK5RAP2 isoform lacking centrosomal localization signal is expressed in developing cortical neurons and this isoform can activate cytoplasmic γTuRCs. In the early stages of dendrite growth, neurons form many short MTs in the cytoplasm (Hasaka et al., 2004, Yamada and Hayashi, 2019), which might be then transported into the elongating dendrite (Sharp et al., 1997), where they undergo further polymerization or being amplified by MT-severing enzymes such as katanin-like 1 (Hatakeyama and Hayashi, 2018). Thus, cytoplasmic nucleation of MTs might be one of the regulatory steps for dendrite growth (Yamada and Hayashi, 2019). Since exclusion of exon 17 does not occur in more than half of the total CDK5RAP2 mRNA of neurons, there remains room to elevate the ratio of exclusion. Artificial manipulation of mRNA splicing with an antisense oligonucleotide, such as those used for Duchenne muscular dystrophy therapy to skip mutated exon of dystrophin mRNA (Matsuo, 2021), can be applied in the future to enhance exon 17 exclusion. This would possibly increase MT-nucleation in neurons and eventually promote dendrite growth.

## Ethical statements

All procedures were conducted according to the Guide for the Care and Use of Laboratory Animals (NIH publications No. 80–23) and the guidelines of the Animal Experiment Committee of Sophia University.

## CRediT authorship contribution statement

Kensuke Hayashi designed and supervised the study. Akari Nakamura performed almost all experiments. Mami Ikeda contributed to the RT-PCR analysis in the early phase of this study and Seina Kusayanagi participated in the immunoprecipitation experiments. All authors read and approved the final manuscript.

## Conflicts of Interest

The authors declared no potential conflicts of interest with respect to the research, authorship, and publication of this article.
